# Are open-access fees undermining science as a public good?

**DOI:** 10.17179/excli2025-8867

**Published:** 2025-10-29

**Authors:** Lucindo Quintans-Júnior, Jodnes Sobreira Vieira, Paulo Ricardo Martins-Filho

**Affiliations:** 1 Laboratory of Neurosciences and Pharmacological Assays (LANEF), Federal University of Sergipe, São Cristóvão, SE, Brazil; 2 Graduate Program in Health Sciences (PPGCS); 3 Investigative Pathology Laboratory (LPI), Federal University of Sergipe, Aracaju, SE, Brazil

## ⁯

The rhetoric of “open science” increasingly masks an economic model that enriches private publishers while exploiting researchers' intellectual work, as Katharine Sanderson (2023[4]) noted. This billion-dollar industry thrives on an uneven logic: governments and funding agencies - particularly in low- and middle-income countries (LMICs) - support research, yet its dissemination often depends on journals demanding unaffordable open-access fees. Scientific dedication is thereby transformed into revenue streams for a few publishers in high-income nations, a concern raised more than a decade ago (Van Noorden, 2013[5]).

Recent practices intensify this imbalance: rapid rejections from “free” open-access journals are followed by invitations to costly titles under the same publisher. Such mechanisms weaken transformative agreements, drain public resources, and consolidate monopolies. For LMICs, the effect is exclusion: scarce budgets are diverted to cover publication charges, and participation in the global scientific dialogue is constrained.

An example of abusive publication fees and researchers' revolt came from *NeuroImage*, one of Elsevier's leading journals. More than 40 renowned scientists, including professors from Oxford, King's College, and Cardiff, resigned en masse from its editorial board in protest against the high publication charges. The group deemed the fee of over £2,700 per article “unethical” and driven by greed, as Elsevier's profit margins exceed 40 %. The editors have since founded a nonprofit journal promoting fair and accessible science (Gogoni, 2023[2]; Fazackerley, 2023[1]). Scientists write for free, reviewers work for free, and society, which already foots the bill for science, is kindly invited to pay once more - this time for the privilege of reading what it has already funded. A perfect business model: all the costs outsourced, all the profits privatized. By charging thousands of dollars for indexing or access, Elsevier and its peers show no commitment to science, but rather to perpetuating a business model that benefits the few while penalizing the many. Obviously, researchers have been courted - and even lured - by predatory journals whose only compass is profit. Some publishers have turned predation into an art: they seduce with the promise of open science, flatter researchers' egos, and, in the same move, cash in with “miraculous” discounts that still leave fees extortionate. It is a bottomless pit - and science, poor thing, is already in it (Quintans-Júnior et al., 2023[3]).

Universities and research centers, especially in chronically underfunded contexts, must urgently embrace alternatives to major publishers. Publishing in reputable journals linked to scientific societies, with rigorous peer review and a focus on advancing knowledge - rather than profit - is one path. Open databases, scientific and institutional repositories also offer solutions. But collective thinking is needed: why not create a large non-profit publisher, supported by consortia such as the BRICS or the European Union, dedicated to open science and social benefit, free from the predatory lobbying of publishing giants?

Thus, if science is to remain a public good, new models are urgent. Publicly funded, collaborative platforms led by LMICs and other under-resourced regions could reduce dependence on commercial publishers and restore the true purpose of open access: equitable knowledge sharing.

## Declaration

### Conflict of interest

The authors declare no conflict of interest.

### Artificial Intelligence (AI) - Assisted Technology

Moreover, an AI tool was used exclusively to refine the English language of the manuscript.
